# Coordinate hypermethylation at specific genes in prostate carcinoma precedes LINE-1 hypomethylation

**DOI:** 10.1038/sj.bjc.6602030

**Published:** 2004-08-03

**Authors:** A R Florl, C Steinhoff, M Müller, H-H Seifert, C Hader, R Engers, R Ackermann, W A Schulz

**Affiliations:** 1 Department of Urology, Heinrich-Heine-University Duesseldorf, Moorenstr 5, 40225 Duesseldorf, Germany; 2 Department Computational Molecular Biology, Max Planck Institute for Molecular Genetics, Ihnestr. 63-73, 14195 Berlin, Germany; 3 Department of Pathology, Heinrich-Heine-University Duesseldorf, Moorenstr. 5, 40225 Duesseldorf, Germany; 4 Center for Biological and Medical Research, Heinrich-Heine-University Duesseldorf, Moorenstr. 5, 40225 Duesseldorf, Germany

**Keywords:** *GSTP1*, *RARB2*, *ASC1*, *SFRP1*, LINE retrotransposons

## Abstract

In prostate carcinoma (PCa) increased DNA methylation (‘hypermethylation’) occurs at specific genes such as *GSTP1*. Nevertheless, overall methylation can be decreased (‘hypomethylation’) because methylation of repetitive sequences like LINE-1 retrotransposons is diminished. We analysed DNA from 113 PCa and 36 noncancerous prostate tissues for LINE-1 hypomethylation by a sensitive Southern technique and for hypermethylation at eight loci by methylation-specific PCR. Hypermethylation frequencies for *GSTP1*, *RARB2*, *RASSF1A*, and *APC* in carcinoma tissues were each >70%, strongly correlating with each other (*P*<10^−6^). Hypermethylation at each locus was significantly different between tumour and normal tissues (10^−11^<*P*<10^3^), although hypermethylation, particularly of *RASSF1A*, was also observed in noncarcinoma tissues. *ASC1* hypermethylation was observed in a subgroup of PCa with concurrent hypermethylation. Hypermethylation of *CDH1, CDKN2A*, and *SFRP1* was rare. LINE-1 hypomethylation was detected in 49% PCa, all with hypermethylation at several loci. It correlated significantly with tumour stage, while hypermethylation was neither related to tumour stage nor Gleason score. Coordinate hypermethylation of several genes may occur early in PCa, with additional hypermethylation events and LINE-1 hypomethylation associated with progression. Hypermethylation allows detection of >82% of PCas. PCa may fall into three classes, that is, with few DNA methylation changes, with frequent hypermethylation, or with additional LINE-1 hypomethylation.

In many human tumours, two different types of alterations of DNA methylation are found, termed ‘hypermethylation’ and ‘hypomethylation’. At specific sites in the genome methylation is increased, typically in CpG islands around the transcriptional start sites of genes silenced in tumours (Baylin and Jones, 2002). Although hypermethylation occurs to some extent during ageing and in preneoplastic tissues, dense methylation of CpG islands is largely tumour-specific and can be exploited for tumour detection. Hypermethylation is frequent in prostate carcinoma (PCa). For instance, hypermethylation of the *GSTP1* gene occurs in >70% of PCa and has been shown to be useful for its detection (Lee *et al*, 1994; Esteller *et al*, 1998; Millar *et al*, 1999; Santourlidis *et al*, 1999; Goessl *et al*, 2001; Jeronimo *et al*, 2001; Nakayama *et al*, 2004). Hypermethylation of this gene is established during the initial stages of PCa development (Brooks *et al*, 1998). Several other genes, such as *APC*, *CDH1*, *CDKN2A*, *RASSF1A*, and *RARB2* (Graff *et al*, 1995; Jarrard *et al*, 1997; Nakayama *et al*, 2001; Liu *et al*, 2002; Maruyama *et al*, 2002; Yamanaka *et al*, 2003), have been reported to be hypermethylated in prostate cancer with various frequencies (reviewed in Schulz and Seifert, 2003). Further genes have been reported as hypermethylated in other human cancers, but have not been studied in PCa. They include the WNT signalling modulator gene *SFRP1* (Suzuki *et al*, 2002) at 8p12, a common region of chromosome loss in PCa (Dong, 2001), and the putative apoptosis regulator gene *ASC1/TMS1* (Conway *et al*, 2000) at 16p12.

An important question is how multiple hypermethylation events relate to tumour progression. One possibility is that genes become successively hypermethylated with carcinoma progression, resulting in an increase in the number of methylation changes during tumour development. A second possibility is that PCa may display patterns of methylation changes related to their biological behaviour, implicating that patterns of DNA methylation changes might be associated with different clinical courses. Two recent studies on PCa have yielded discrepant results on these issues (Maruyama *et al*, 2002; Yamanaka *et al*, 2003). A third possibility is that carcinomas fall into different subclasses, one of which is characterised by a high frequency of methylation alterations. A precedent is the CIMP+subclass of colon cancers (Toyota *et al*, 2000).

In spite of hypermethylation at specific sites, the overall methylcytosine content in human tumour cells is often decreased (‘global’ or ‘genome-wide’ hypomethylation) because methylation of repetitive sequences such as retroelements and CpG-rich satellites, which contain most of the methylcytosine in normal somatic cells, is diminished (Robertson, 2001; Baylin and Jones, 2002; Ehrlich, 2002). In particular, LINE-1 retrotransposons comprise 17% of the human genome and contain a disproportionate fraction of methylcytosine. The methylation status of LINE-1 sequences therefore provides a good indicator of global hypomethylation in tumour cells. Moreover, LINE-1 hypomethylation may lead to re-expression of individual elements and promote genomic instability (Florl *et al*, 1999; Schulz *et al*, 2002). In PCa, overall DNA methylation and LINE-1 methylation are decreased most consistently in metastatic cases (Bedford and van Helden, 1987). The precise relationship between specific hypermethylation events and global hypomethylation in PCa has not been studied.

Here, we have investigated 113 PCas for hypermethylation at eight loci by methylation-specific PCR (MS-PCR) and for LINE-1 methylation as an indicator of global hypomethylation by Southern blot hybridisation. The results suggest that with regard to DNA methylation alterations PCas may fall into different subclasses.

## MATERIALS AND METHODS

### Tissues

PCa specimens were obtained between 1993 and 2002, almost all by radical prostatectomy. Carcinoma and morphologically normal areas of the prostate were identified, and specimens collected by a pathologist, rapidly frozen in liquid nitrogen and stored at −80°C. Since several micrograms of high molecular weight DNA were required for LINE-1 hypomethylation analysis and multiple, repeated MS-PCR assays, no microdissection was performed. Immediately after surgical removal, prostates were sectioned by an experienced pathologist. Tumour and matched tumour-free specimens were only collected (i) when tumours were grossly apparent in the peripheral zone and could be unequivocally identified by their characteristic yellow or orange-yellow colour and (ii) when the transition zone was macroscopically free of tumour burden. Representative samples of 3 mm maximal diameter of tumour and tumour-free tissue specimens were collected, immediately snap frozen in liquid nitrogen and stored at −80°C. Noncancerous tissue samples were taken from areas of the transition zone as far away as possible from the grossly apparent tumour (i.e. in general from the transition zone of the contralateral lobe). Macroscopic separation between tumour and nontumorous tissues was histologically verified by analysing tissue specimens immediately adjacent to the specimens collected for methylation analysis. TNM classification was performed according to the guidelines of the International Union Against Cancer (UICC) from 1997. Of 113 PCa tissues, 48 carcinomas were staged as pT2, 59 as pT3, and five as pT4, one tumour was staged as pT1. Lymph node metastases were present in 21 patients, and distal metastases were detected in one patient. Four tumours had Gleason scores <5, 77 of 5–7, and 32 of >7. The patients' mean age was 66 years, ranging from 46 to 79 years. Overall, 36 carcinoma-free tissues were investigated, of which seven were obtained by adenomectomy or cystoprostatectomy for bladder cancer. The study was approved by the ethics committee of the Heinrich-Heine University medical faculty.

### Cell lines

The bladder carcinoma cell line T24 was used as a positive control for methylation of *CDH1, CDKN2A*, and *SFRP1.* The PCa cell line LNCaP was used as a positive control for methylation of *APC*, *ASC1*, *GSTP1*, *RARB2*, and *RASSF1A*.

### DNA extraction

High molecular weight genomic DNA from tissue, cell lines, and whole blood was isolated using the blood and cell culture DNA kit (QIAGEN, Hilden, Germany). Frozen tissues were crushed to a fine powder with a mortar and pestle before extraction.

### Methylation-specific PCR

In all, 1 *μ*g of DNA from each sample was bisulphite-treated using the CpGenome™ DNA Modification Kit (Oncor, Heidelberg, Germany) and 80 ng each were used in separate PCR reactions with primer pairs specific for methylated or unmethylated DNA (Supplementary Table). PCR reactions were performed in a volume of 50 *μ*l containing 1.5 mM MgCl_2_, 150 *μ*M dNTPs, 0.6 *μ*M of each primer, and 1.25 U HotStar Taq polymerase (QIAGEN). The initial denaturing step at 95°C for 15 min was followed by 35–38 cycles each consisting of a denaturing step at 94°C for 30 s, primer annealing at 53–65°C (Supplementary Table) for 30 and a 45 s elongation step at 72°C. The final 72°C period was extended to 8 min. PCR products were separated on 2% agarose gels and visualised by ethidium bromide. Results were scored by two independent observers. All assays were repeated at least twice with independent bisulphite treatments and concordant results.

For bisulphite sequencing PCR products were subcloned into the PCR-4-TOPO vector (Invitrogen, Groningen, NL, USA) and several clones each were sequenced by standard methods.

### LINE-1 hypomethylation analysis

Aliquots from the same DNA were employed to detect LINE-1 hypomethylation. In all, 1 *μ*g of high molecular weight DNA each was extensively digested with the methylation-sensitive restriction enzyme *Hpa*II or its methylation non-sensitive isoschizomer *Msp*I, separated on agarose gels, blotted and hybridised with a ^32^P-labelled specific LINE-1 probe. After *Hpa*II digestion, decreased methylation of LINE-1 sequences results in the appearance of new bands in the 1.0–4.0 kb range on Southern blots whose intensities relative to the *Msp*I signals, after correction for unequal loading of the two lanes, can be used to quantitate hypomethylation. This method detects as little as 1% hypomethylation (Florl *et al*, 1999; Santourlidis *et al*, 1999). For categorisation, 0–4% hypomethylation was considered unchanged (0), 5–12% as moderate (1) and >12% as strong (2), slightly modified from a previous report (Schulz *et al*, 2002).

### Mathematical procedures

Statistical analyses were performed using S-Plus 4.5. Professional, Release 2, MathSoft Inc., and MatLab, version 6.1.0.450 (R12.1), The Math Works, Inc. For CART analysis no weights were used. Hierarchical clustering was calculated based on Euclidean distance and average linkage.

## RESULTS

**Figure 1:**
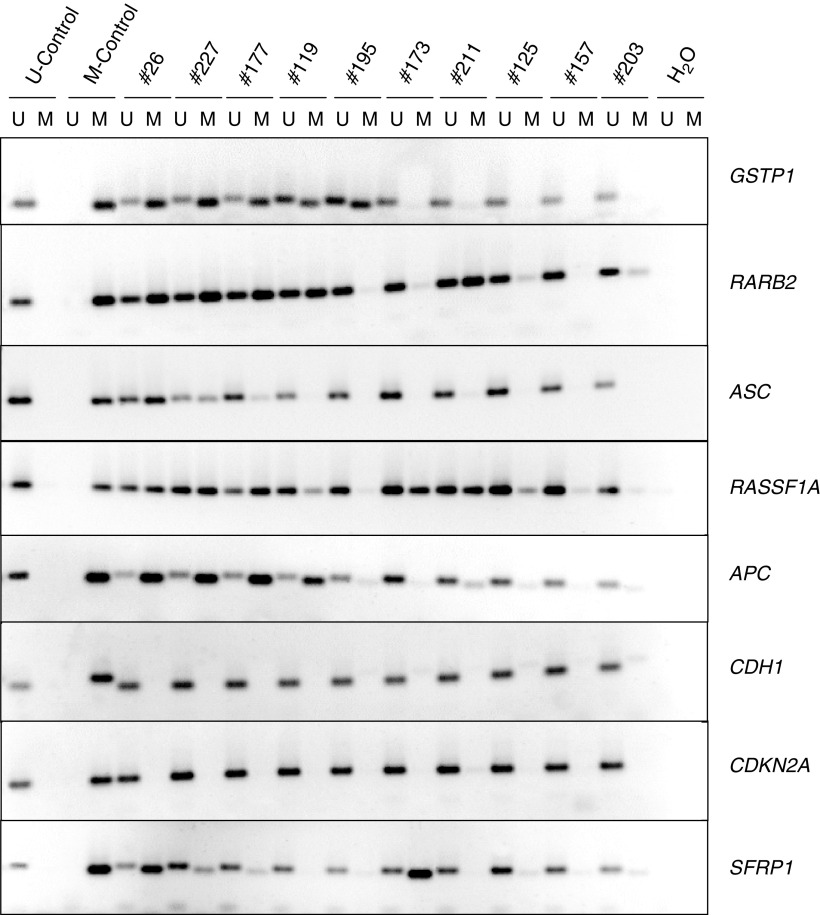
Methylation specific PCR. Representative examples of MS-PCR analyses for the methylated (M) and the unmethylated (U) forms of eight genes in prostate cancer. Selected tumour samples with decreasing frequency of hypermethylation events from left to right. The bladder cancer cell line T24 was used as a positive control for *CDH1, CDKN2A*, and *SFRP1* hypermethylation. The prostate carcinoma cell line LNCaP was used for *ASC1, APC*, *GSTP1*, *RARB2*, and *RASSF1A* hypermethylation (M control). Leukocyte DNA from a female without cancer was used as a negative control (U control).

Hypermethylation was investigated at eight loci in 113 PCa specimens using published validated MS-PCR methods (Supplementary Table). Six of these loci had previously been reported to be hypermethylated in prostate cancers, whereas *ASC1* and *SFRP1* had been reported as frequently hypermethylated in other cancers, but not been studied in prostate cancer before. They were chosen to be located on different chromosomes (with the exception of *RARB2* and *RASSF1A*), and to represent a wide range of established or presumed functions. In each MS-PCR assay controls were carried through the procedure, that is, DNA from a cell line with hypermethylation of the respective gene as a positive control and DNA from normal blood leukocytes as a negative control (Figure 1

).

**Figure 2:**
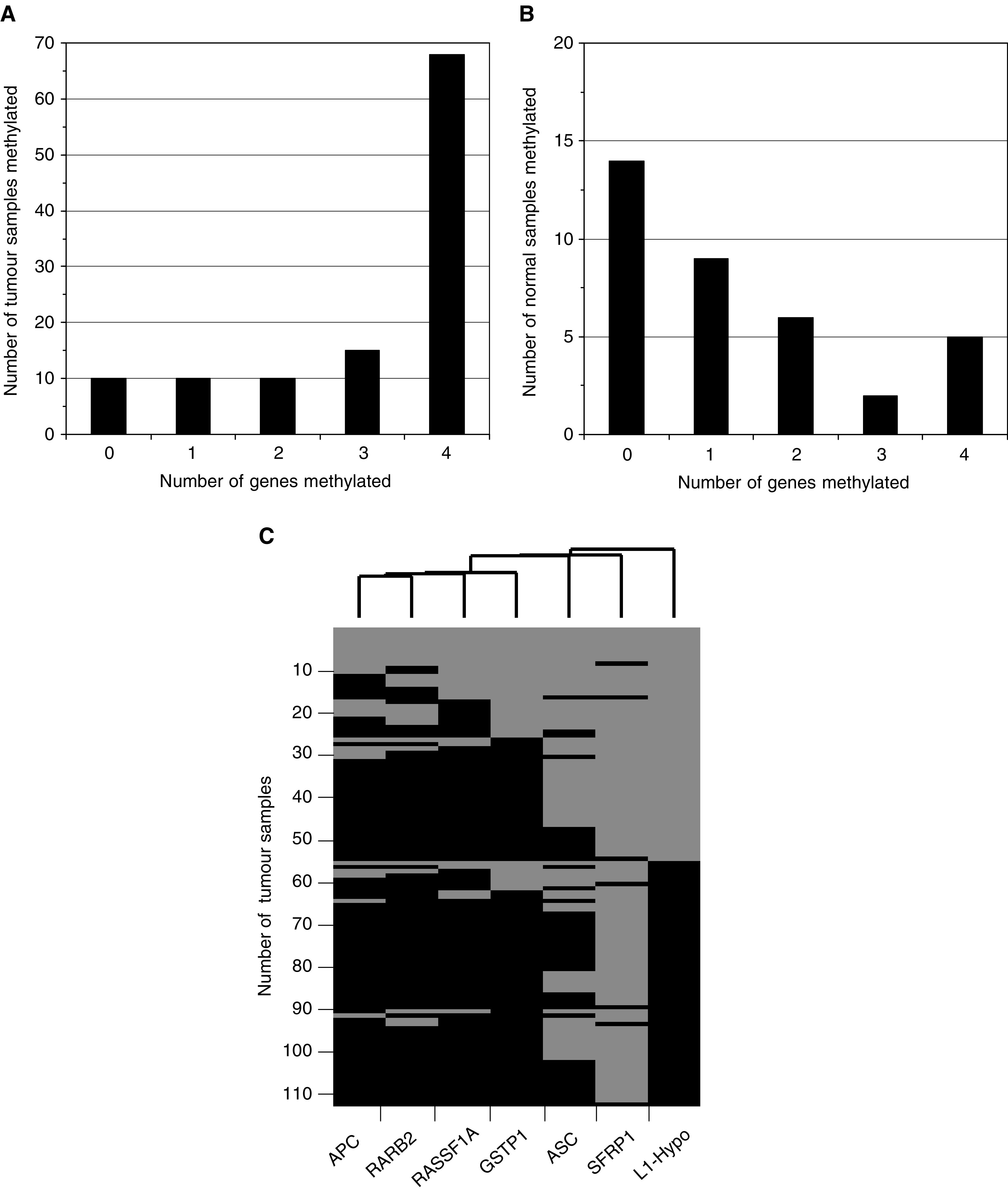
Hypermethylation in prostate cancer and noncancerous tissues. (**A**) Number of cases with hypermethylation detected in the indicated number of genes (*APC*, *GSTP1*, *RARB2*, and *RASSF1A*) in PCa tissues. (**B**) Number of cases with hypermethylation detected in the indicated number of genes (*APC*, *GSTP1*, *RARB2*, and *RASSF1A*) in prostate tissues morphologically free of carcinoma. (**C**) Cluster analysis of *APC*, *ASC1, GSTP1*, *RARB2*, *RASSF1*, and *SFRP1* hypermethylation in PCa tissues. Black: hypermethylated; grey: unmethylated. LINE-1 hypomethylation is also shown. Black: hypomethylation >4%; grey: normal methylation.

The eight loci fell into three groups with regard to hypermethylation in PCa tissues. First, for *APC, GSTP1*, *RARB2*, and *RASSF1A* hypermethylation was frequent and found in 78, 79, 70, and 78% of the cases, respectively. Methylation at each of the four loci strongly correlated highly significantly with that at any of the other loci. Thus, 68 (60%) PCa specimens exhibited methylation at all four loci, while in 10 (9%) cases none of the loci was hypermethylated (Figure 2A

). In addition, 10 PCa specimens displayed methylation in only one gene, most often in *RASSF1A*, followed by *APC*. This distribution is highly unlikely to arise by chance (*P*<10^−6^) and suggests a coordinate process. Secondly hypermethylation of *CDH1*, *CDKN2A*, and *SFRP1*, was rare in PCa tissues (<5% of the specimens), although they were hypermethylated in cancer cell line controls (Figure 1). This result was not due to lack of sensitivity, since dilution experiments with T24 cell line DNA showed that the MS-PCR method detected ≈1% hypermethylated alleles for each gene (data not shown). Moreover, the data for *SFRP1* were confirmed by bisulphite sequencing in several positive and negative samples (data not shown). Thirdly, hypermethylation of *ASC1* was found in 40% of PCa. Interestingly, these also displayed hypermethylation in at least three of the genes *APC, GSTP1, RARB2*, and *RASSF1A.* Thus, the PCa specimens with *ASC1* hypermethylation formed a precise subgroup of those with concomitant hypermethylation of these four genes (Figure 2C).

Many noncarcinoma prostate specimens were free of hypermethylation, but some yielded signals in MS-PCR analyses. In these, the most frequently hypermethylated gene was *RASSF1A* (53%), followed by *APC* (36%), while hypermethylation of GSTP1 (19%) and RARB2 (17%) was infrequent. This is the same order as in tumour tissues with hypermethylation of only a single gene. Importantly, concomitant hypermethylation of several genes was much rarer in noncarcinoma than in carcinoma tissues (Figure 2B).

**Figure 3:**
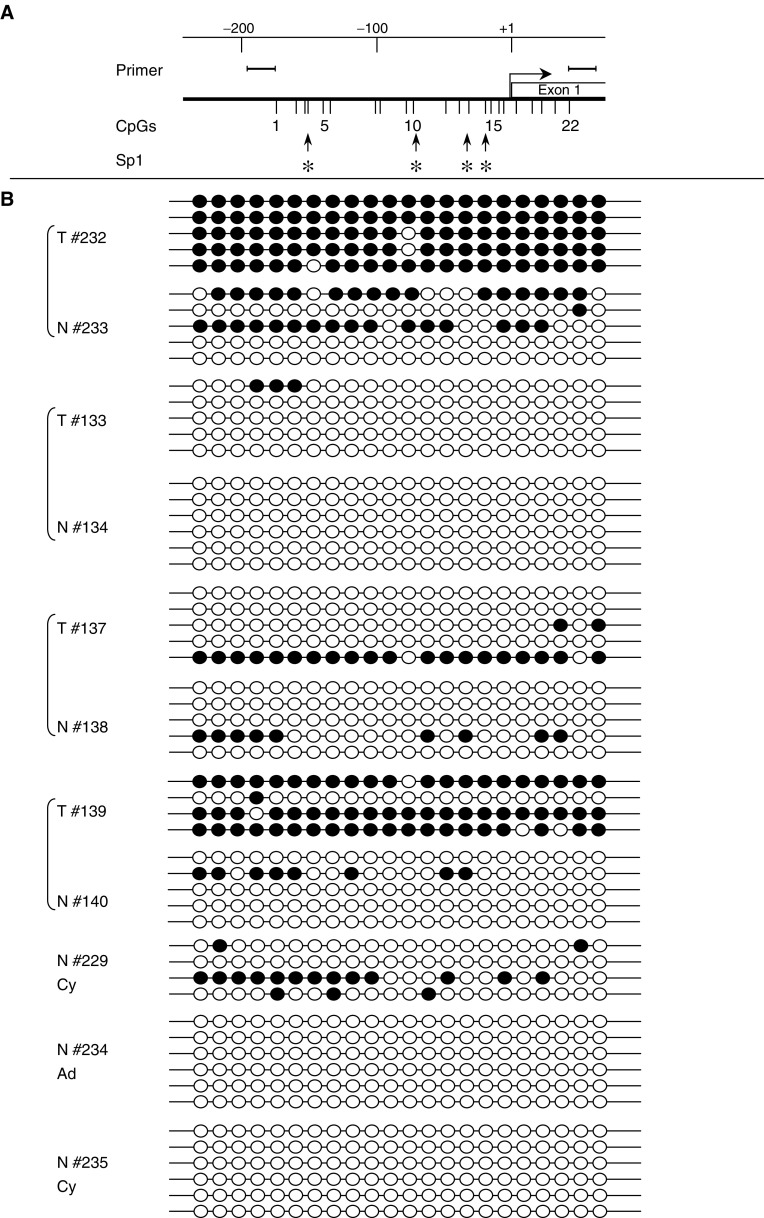
Bisulphite methylation analysis of *RASSF1A*. Methylation status of the *RASSF1A* promoter CpG island in paired prostate carcinoma (T) and adjacent normal (N) tissue samples (#232/233, #133/134, #137/138, #139/140) as well as carcinoma-free prostate specimens obtained during adenomectomy for benign prostate hyperplasia (AD, #234) or cystoprostatectomy for bladder cancer (Cy, #229 and #235). Each circle depicts an individual CpG site. Black: methylated site, white: unmethylated site. At the top of the figure, the localisation of the 22 investigated sites in the RASSF1A promoter are indicated. Several potential binding sites for Sp1 are marked by asterisks.

Since *RASSF1A* was most frequently methylated in noncarcinoma tissues, its methylation pattern was compared between several normal and tumour specimens by bisulphite sequencing (Figure 3

). In tumour tissues (T#232 and T#139), individual *RASSF1A* alleles were often continuously methylated. In the corresponding normal tissues (N#233 and N#140), methylation of individual alleles was less dense. Tumour tissues reacting weakly positive in MS-PCR (T#133 and T#137) showed patchy methylation and methylation was rare in the corresponding normal tissues (N#134 and N#138). One prostate sample without carcinoma from an adenomectomy for benign hyperplasia and one from a cystoprostatectomy for bladder cancer were devoid of methylation (N#234, N#235). A further carcinoma-free prostate specimen (N#229) obtained by cystoprostatectomy for bladder cancer showed patchy methylation in *RASSF1A*.

Accordingly, among the four frequently hypermethylated genes, hypermethylation of *RASSF1A* discriminated least well between normal and tumour tissues. However, even for this gene the frequency of hypermethylation was significantly different between carcinoma and noncarcinoma tissues (*p*(*χ*^2^)=10^−3^). The significance levels for other loci were 3 × 10^−5^ (*APC*), 1.2 × 10^−6^ (*GSTP1*), and 1.2 × 10^−10^ (*RARB2*). In a CART analysis, optimal separation between tumour and normal tissues was achieved using *RARB2* plus *GSTP1* hypermethylation. This procedure classified 82% of all cancers correctly, while inclusion of further loci improved detection marginally to 83% (i.e. by one case). The specificity of the combination *RARB2* plus *GSTP1* hypermethylation was 83%, which was identical to that of *RARB2* hypermethylation alone, similar to that of *GSTP1* (81%), and considerably higher than those of *APC* (61%) and *RASSF1A* (47%).

**Figure 4:**
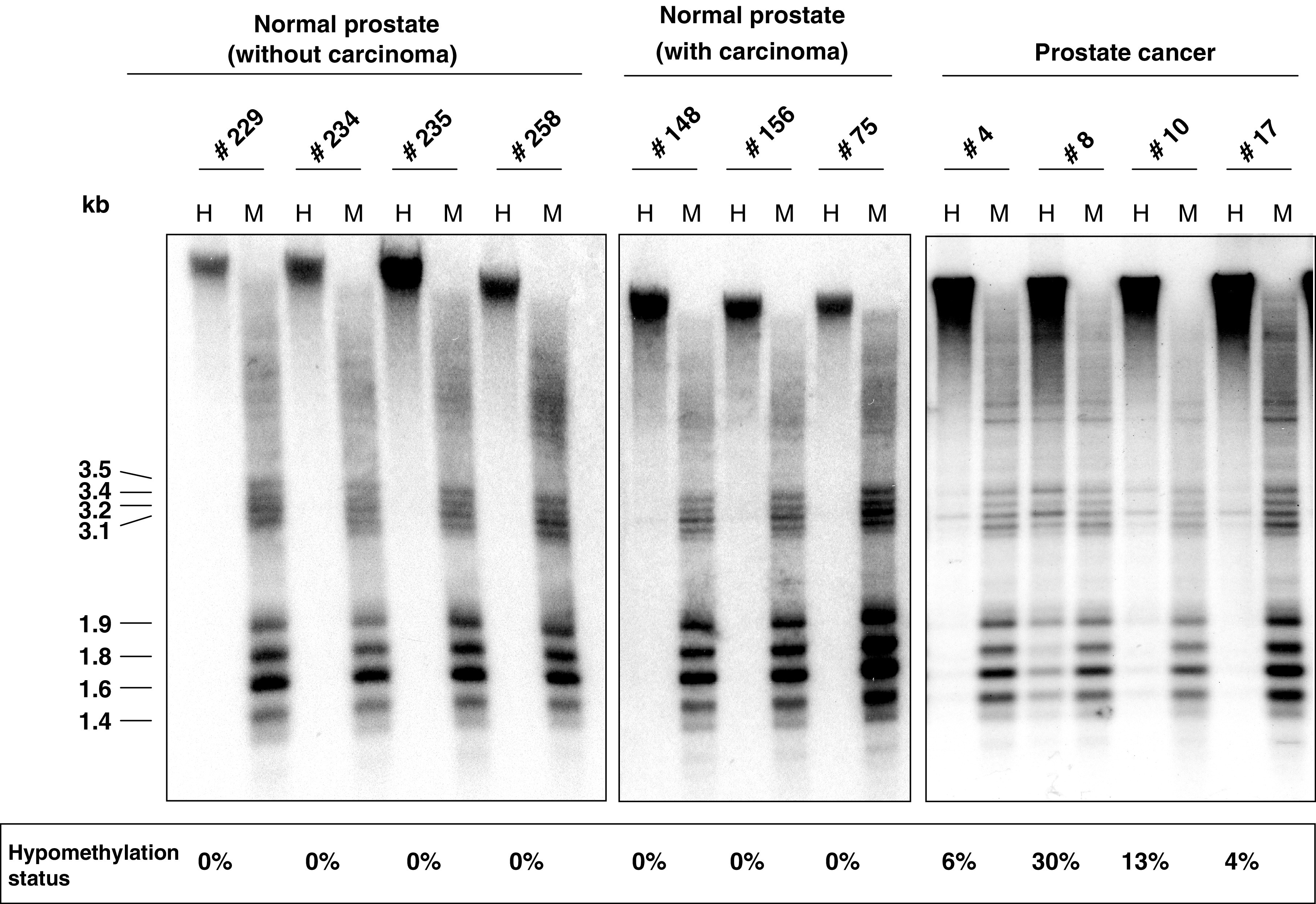
Southern blot analysis of LINE-1 hypomethylation in prostate and prostate carcinoma tissues. DNA isolated from prostates carrying no carcinoma (left), carrying carcinoma (center), and from prostate carcinomas (right) was analysed for LINE-1 hypomethylation as described in the Materials and Methods section. H: *Hpa*II digestion; M: *Msp*I digestion. Hypomethylation is detectable by the appearance of bands in the 1.4–3.5 kb range in the H lane. Results of densitometric quantification (see Materials and Methods for details) are depicted at the bottom of the figure.

**Figure 5:**
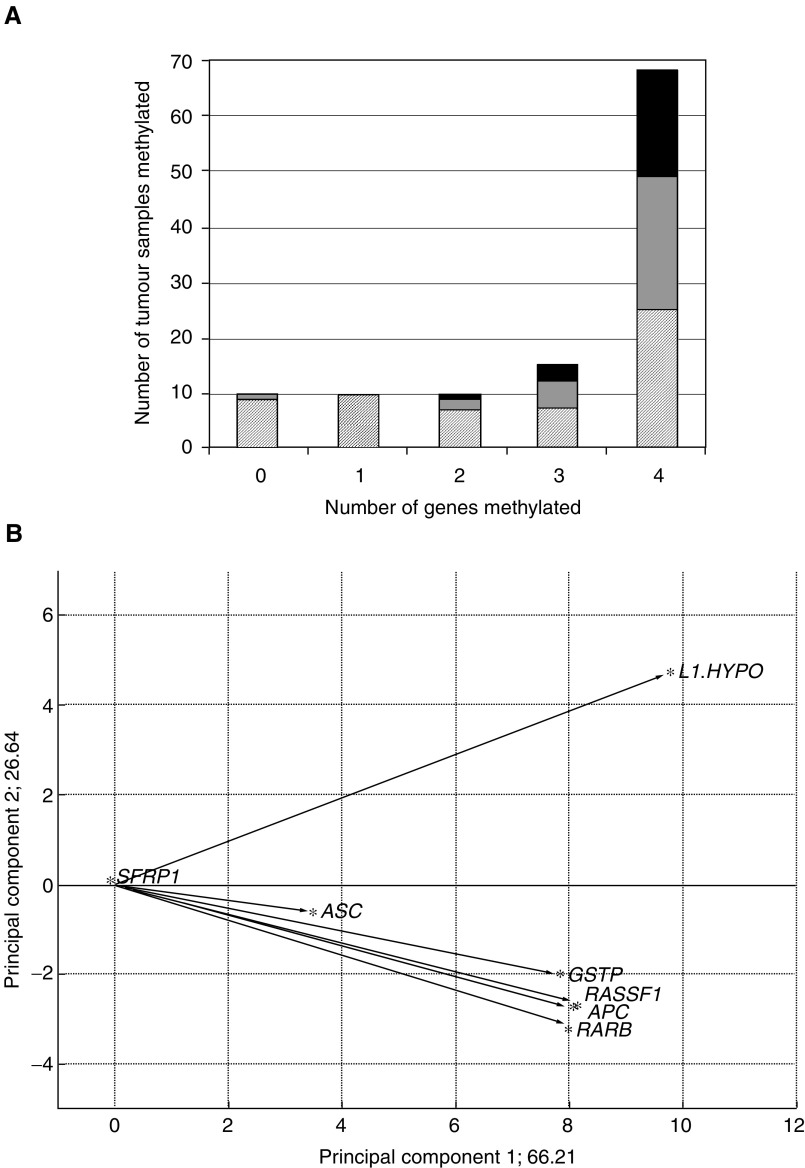
Relationship of LINE-1 hypomethylation to gene hypermethylation in prostate carcinoma samples. (**A**) Number of cases with hypermethylation detected in the indicated number of genes in prostate carcinoma tissues (*APC*, *GSTP1*, *RARB2*, and *RASSF1A*) and lack of hypomethylation (black bars), moderate hypomethylation (grey bars), and pronounced hypomethylation (striped bars). (**B**) Principal component analysis of the tumour methylation data set for the factors LINE-1 hypomethylation (L1-HYPO) as well as *APC*, *ASC1*, *GSTP1*, *RARB2*, *RASSF1A*, and *SFRP1* hypermethylation. Plot of the first two components from principal component analysis. The first component explains 66.2% of the variance, the second 26.6%. Asterisks represent the factors, while arrows demonstrate the direction from the origin.

Hypomethylation of LINE-1 sequences (Figure 4

) was observed in 49% of PCa (Figure 5A

). In 23 specimens pronounced hypomethylation (defined as >12%) was observed, while 33 displayed moderate hypomethylation (defined as 5–12%). LINE-1 hypomethylation correlated with hypermethylation of each of the genes *RARB2*, *RASSF1A*, *GSTP1*, and *APC* (1.7 × 10^−6^ <*p*(*χ*^2^) <3.2 × 10^−5^). Nevertheless, hypermethylation and hypomethylation were not entirely concordant, because many cases with hypermethylation in three or four genes lacked LINE-1 hypomethylation (Figure 5A). Thus, like *ASC1* hypermethylation, LINE-1 hypomethylation was essentially restricted to a subgroup of the cases with concomitant hypermethylation of the four genes (Figure 2C). *ASC1* hypermethylation and LINE-1 hypomethylation correlated closely with each other (*p*(*χ*^2^)=6.0 × 10^−4^), but the subgroups were not identical. These relationships are illustrated by principal component analysis (Figure 5B), which yielded almost identical vectors for hypermethylation of each of the four genes, but separation of the vectors representing *ASC1* hypermethylation in the first component and LINE-1 hypomethylation in the second component. No significant LINE-1 hypomethylation was seen in any noncancer prostate specimen analysed (Figure 4).

None of the more frequent hypermethylation events, including *ASC1* hypermethylation, were significantly related to tumour stage or Gleason score. All six cases with *SFRP1* hypermethylation were staged as pT2 and this rare change was therefore significantly related to tumour stage (*P*=0.02). The number of hypermethylation events was unrelated to clinical parameters (Table 1

**Table 1:** Summary of MS-PCR parameters

		
		**Gleason score**		
**(a)**	**# of genes hypermethylated**	**1–4**	**5–7**	**8–10**
*P*=0.1781	0–1	3	28	14
	2–3	0	15	10
	4	1	34	8
				
		**Tumour stage**		
		**pT1–2**	**pT3–4**	
*P*=0.2024	0–1	24	21	
	2–3	10	15	
	4	15	28	
				
		**Lymph node status**		
		N0	N+	
*P*=0.4817	0–1	39	6	
	2–3	19	6	
	4	34	9	
				
				
		**Gleason score**		
**(b)**	**Hypomethylation**	**1–4**	**5–7**	**8–10**
*P*=0.5937	0–3%	1	40	14
	4–12%	2	24	9
	>12%	1	13	9
				
		**Tumour stage**		
		**pT1–2**	**pT3–4**	
*P*=0.04187	0–3%	29	26	
	4–12%	15	20	
	>12%	5	18	
				
		**Lymph node status**		
		**N0**	**N+**	
*P*=0.08172	0–3%	47	8	
	4–12%	30	5	
	>12%	15	8	

For each gene the primer sequences used, the annealing temperatures, and the number of cycles are indicated. M=methylated-specific primers; U=unmethylated-specific primers; BS=primers used for bisulphite sequencing.

). LINE-1 hypomethylation was significantly more frequent in higher stage carcinomas (*P*=0.04) and tended to be more pronounced in lymph node-positive PCa (*P*=0.08).

## DISCUSSION

Hypermethylation of *GSTP1* is a promising marker for detection of PCa (Lee *et al*, 1994; Esteller *et al*, 1998; Millar *et al*, 1999; Santourlidis *et al*, 1999; Goessl *et al*, 2001; Jeronimo *et al*, 2001). Different studies have reported somewhat different proportions of positive cancers, likely due to differences in methodology as well as to differences between the patient populations investigated. Here, using a sensitive MS-PCR method, we found 70% of carcinoma tissues from a Central European population to display *GSTP1* hypermethylation with rare methylation in adjacent normal tissues, supporting its value as a marker. In the same specimens, three further genes, that is *APC*, *RARB2*, and *RASSF1A*, proved to be hypermethylated at similarly high frequencies, and also discriminated well between tumour and normal tissue. A combination of several genes may be optimal for discrimination and could be used as described here or in combination with quantitative methylation analyses (Jeronimo *et al*, 2001). In our data set, optimal separation between carcinoma and normal tissues was achieved by *GSTP1* and *RARB2* hypermethylation, identifying >80% tumour samples correctly. The specificity of detection by this combination was 83% and thus not exceedingly high. However, as the noncarcinoma specimens were mostly taken from prostates that harboured cancers, this level of specificity is likely an underestimate (see discussion below).

The fact that two of these four genes are sufficient to detect all carcinomas with hypermethylation follows from the strong correlation between hypermethylation of these individual genes. If each hypermethylation event occurred independently at the observed frequencies (70–79%), most carcinomas should display hypermethylation in two or three genes and <1/113 should lack hypermethylation. Therefore, we observed more tumours with simultaneous hypermethylation of all four genes as well as more tumours without hypermethylation at any of the loci than expected from a stochastic process (cf Figure 2). This suggests that hypermethylation occurs in a coordinate manner in most cases and, conversely, that a smaller subset of PCas is not prone to hypermethylation. It may be difficult to distinguish PCa in this subset reliably from normal or ageing prostate by DNA methylation analysis. There is so far little indication for major differences in clinical course between the hypermethylation-prone and rare-hypermethylation groups (Supplementary Table). This fits the emerging consensus that deregulation of DNA methylation can be as effective at driving tumour development and progression as chromosomal instability or base repair deficiency (Baylin and Jones, 2002).

The strongly coordinated hypermethylation of the above four genes is remarkable in view of the low hypermethylation rate detected in three other genes that are often hypermethylated in other cancers. Our data on CDKN2A/p16 are in accordance with other reports on a low percentage of hypermethylation of this gene in PCa (Jarrard *et al*, 1997; Maruyama *et al*, 2002). The reported frequencies of *CDH1* hypermethylation in PCa differ widely between published studies (Graff *et al*, 1995; Woodson *et al*, 2003). Detailed analyses have shown hypermethylation patterns in *CDH1* to be very variable (Graff *et al*, 2000), which may partly explain these differences. At any rate, such a variability would preclude a use in diagnostics. Hypermethylation of *SFRP1* was found in other cancers (Suzuki *et al*, 2002), but had not been studied before in PCa. This gene was of particular interest because of its location in a chromosomal region with a high frequency of allelic loss in PCa (Dong, 2001). In this regard, our data indicate that the gene may become inactivated by hypermethylation in individual cases of PCa. Our data suggest a more significant involvement of *ASC1*. This gene has previously been identified as being silenced by hypermethylation in breast carcinoma and is thought to be involved in the regulation of apoptosis (Conway *et al*, 2000). Its precise role in PCa certainly deserves further investigation.

The comparison of the two groups of genes – *APC*, *GSTP1*, *RARB2*, and *RASSF1A vs CDH1*, *CDKN2A*, and *SFRP1* – suggests no obvious explanation why they should become hypermethylated at such different frequencies. Specifically, *GSTP1*, *RARB2*, *APC*, and *RASSF1A* are not known to function in a common pathway or – with the exception of *RARB2* and *RASSF1A* – reside on the same chromosome. Our data call for a mechanistic explanation of this unexpected specificity.

In the course of PCa, the origin of *GSTP1* hypermethylation has been traced to late preneoplastic stages like high-grade prostatic intraepithelial neoplasia (Brooks *et al*, 1998; Yamanaka *et al*, 2003) and proliferative inflammatory atrophy lesions (Nakayama *et al*, 2003; Yamanaka *et al*, 2003). If *GSTP1* hypermethylation is associated with the onset of prostate carcinogenesis, it cannot be related to tumour stage or Gleason score, as observed here and by others (Yamanaka *et al*, 2003). Furthermore, because *GSTP1* hypermethylation correlated strongly with that of three other genes, their hypermethylation may also occur at early stages of cancer development. Moreover, our data suggest that hypermethylation of *RASSF1A* might begin in preneoplastic tissue. Hypermethylation of *RASSF1A* was most frequently found in normal tissue from carcinoma-carrying prostates, and *RASSF1A* was the locus most often hypermethylated without hypermethylation at other loci. In addition, *RASSF1A* methylation on individual alleles was denser in carcinoma than in normal prostate tissue (Figure 3). This observation is very reminiscent of findings in the colon. There, some genes are only hypermethylated in colon carcinoma, whereas others start to become hypermethylated in ageing and preneoplastic colon. The patchy methylation in ageing tissue becomes intensified in tumour cells (Issa, 2000). Our data suggest that *RASSF1A* may belong to this class in prostate tissue.

The findings on RASSF1A raise the question of how to interpret hypermethylation events observed in normal-appearing prostate tissue. In some cases, hypermethylation might be derived from minimal carcinoma infiltrates not detected on adjacent histological sections, particularly, in the few cases where hypermethylation of several genes was observed (Figure 2B). However, there were several differences between hypermethylation detected in normal and carcinoma tissues. First, the order of the frequencies for different genes was different. *GSTP1*, *RARB2*, *APC*, and *RASSF1A* were each hypermethylated in 70–80% of carcinoma tissues, whereas the order in noncarcinoma tissues was *RASSF1A*>*APC*>*GSTP1*=*RARB2*. Secondly, concomitant hypermethylation was rare in morphologically normal tissues, but was the rule in carcinomas (Figure 2). Thirdly, the methylation pattern in *RASSF1A* was always patchy in non-neoplastic tissues, whereas dense methylation was typically seen in carcinomas (Figure 3). Taken together, these results might argue for age-associated hypermethylation in the prostate like that reported for other organs (Issa, 2000). Hypermethylation in the prostate might help to establish a preneoplastic state that disposes to carcinoma development. Indeed, if the silencing of *GSTP1* by hypermethylation was to promote the initial development of PCa by inducing an increased sensitivity to electrophilic carcinogens, as postulated by others (Nakayama *et al*, 2004), one would have to expect methylation changes to actually precede the development of morphological changes.

Our finding of a lack of correlation between DNA hypermethylation and tumour stage and Gleason grade in the prostate is in good agreement with a study of Japanese patients (Yamanaka *et al*, 2003), but differs from the results in an American cohort (Maruyama *et al*, 2002) in which the number of methylated genes increased with tumour stage and Gleason score. The reasons for these differences may thus be population-related, or may reflect methodical differences. For instance, the study reporting a good correlation of hypermethylation with stage and grade detected *GSTP1* hypermethylation in only 36% of PCa tissues, which is by far the lowest rate reported in the literature.

In our hands, LINE-1 hypomethylation represents the methylation change best related to histopathological parameters of PCa. In previous studies, global DNA hypomethylation was found to be particularly pronounced in specimens from androgen-refractory carcinomas (Bedford and van Helden, 1987; Schulz *et al*, 2002). Accordingly, the present study showed LINE-1 hypomethylation to be more prevalent in high stage and lymph-node positive PCas. LINE-1 hypomethylation obviously characterises a set of PCas within the subclass of those with frequent hypermethylation (Figures 2C and 4) and is associated with higher stage disease. The most straightforward explanation for this finding is that LINE-1 hypomethylation in PCa is a secondary event during progression following DNA hypermethylation. As a corollary, the mechanisms causing DNA hypermethylation at specific sites and DNA hypomethylation of repetitive sequences are likely distinct, as also reported for Wilms tumours (Ehrlich *et al*, 2002). Another change occurring during progression may be *ASC1* hypermethylation, which was also found in a precise subgroup of the hypermethylation-prone cancers, but apparently not the same one exhibiting LINE-1 hypomethylation. However, no significant relationship to stage or grade was found for this change.

Our study therefore suggests a distinction of three subclasses of PCa, one with few DNA methylation changes, one with hypermethylation of several genes, and one with additional hypomethylation of repetitive sequences. This last subclass may be over-represented among high-stage PCa (*P*=0.086 in the present study according to Kruskal–Wallis test). The distinction between these three subclasses might be useful for further development of methylation-based diagnosis of PCa.

Finally, regarding the functional implications of our findings, hypermethylation of promoter sequences is established as a mechanism of silencing of tumour suppressor genes in human cancers (Baylin and Jones, 2002). The *GSTP1* gene is frequently hypermethylated in prostate cancers, but is a less than ideal candidate for a tumour suppressor (reviewed by Nakayama *et al*, 2004). Its loss of function may lead to increased sensitivity against certain electrophilic compounds and perhaps decreased apoptosis, which could promote carcinogenesis in the prostate. The finding in the present study that hypermethylation of *GSTP1* occurs in a coordinate manner with that of other genes raises the interesting possibility that *GSTP1* hypermethylation might be a bystander effect of the inactivation of a gene more directly involved. Specifically, all three other genes found to be coordinately hypermethylated with *GSTP1* in this study, *APC*, *RARB2*, and *RASSF1A*, are established as functionally important in other cancers. Regarding the functional implications of hypomethylation, it has previously been reported to be almost ubiquitous in metastatic PCa (Bedford and van Helden, 1987, Schulz *et al* 2002), in accord with a significant correlation of LINE-1 hypomethylation with tumour stage in the present study. Moreover, LINE-1 hypomethylation was found to be significantly correlated with chromosomal instability (Schulz *et al*, 2002). It is not known, however, whether genome-wide hypomethylation causes chromosomal instability, although there is some experimental support for this idea (Eden *et al*, 2003).
