# Supplementary material for: Coordinate hypermethylation at specific genes in prostate carcinoma precedes LINE-1 hypomethylation
Source: Br J Cancer. 2004 Aug 3;91(5):985–94. doi: 10.1038/sj.bjc.6602030 (PMC2409889; doi:10.1038/sj.bjc.6602030)
Supplement: Supplementary Table [file 91-6602030x1.doc]

Table 2

| **Gene** | **Gene location** | **Forward primer (5’ > 3’)** | **Reverse primer (5’ > 3’)** | **Temp. (°C)** | **Cycle no.** | **Reference** |
| --- | --- | --- | --- | --- | --- | --- |
| *GSTP1* | 11q13 | **M**: ttcggggtgtagcggtcgtc **U**: gatgtttggggtgtagtggttgtt | **M**: gccccaatactaaatcacgacg **U**: ccaccccaatactaaatcacaaca | 59 | 35 | Esteller et al. |
| *RASSF1A* | 3p21 | **M**: gggttttgcgagagcgcg **U**: ggttttgtgagagtgtgtttaG **BS**: aaggagggaaggaagggtaagg | **M**: GCTAACAAACGCGAACCG **U**: CACTAACAAACACAAACCAAAC **BS**: caactcaataaactcaaactcc | 64 59 58 | 38 | Burbee et al. |
| *APC* | 5q21 | **M**: tattgcggagtgcgggtc **U**: gtgttttattgtggagtgtgggtt | **M**: tcgacgaactcccgacga **U**: ccaatcaacaaactcccaacaa | 64 60 | 35 | Tsuchiya et al. |
| *CDH1* | 16q22 | **M**: ttaggttagagggttatcgcgt **U**: taattttaggttagagggttattgt | **M**: taactaaaaattcacctaccgac **U**: cacaaccaatcaacaacaca | 57 53 | 35 32 | Herman et al. |
| *CDKN2A* | 9p21 | **M**: ttattagagggtggggcggatcgc **U**: ttattagagggtggggtggattgt | **M**: gaccccgaaccgcgaccgtaa **U**: caaccccaaaccacaaccataa | 65 60 | 35 | Herman et al. |
| *RARB2* | 3p24 | **M**: tcgagaacgcgagcgattcg **U**: ttgagaatgtgagtgatttga | **M**: gaccaatccaaccgaaacga **U**: aaccaatccaaccaaaacaa | 56 54 | 35 37 | Virmani et al. |
| *SFRP1* | 8p12-p11.1 | **M**: TGTAGTTTTCGGAGTTAGTGTCGCGC **U**: GTTTTGTAGTTTTTGGAGTTAGTGTTGTGT **BS**: tggttttgttttttaaggggtgttgagt | **M**: CCTACGATCGAAAACGACGCGAACG **U**: CTCAACCTACAATCAAAAACAACACAAACA **BS**: TCCTACCRCAAACTTCCAAAAACCTCC | 64 60 60 | 37 | Suzuki et al. |
| *ASC1* | 16p11.2-12.1 | **M**: ttgtagcggggtgagcggc  **U**: ggttgtagtggggtgagtggt | **M**: aacgtccataaacaacaacgcg **U**: caaaacatccataaacaacaacaca | 58 | 36 | Conway et al. |

Sources: Burbee DG, Forgacs E, Zochbauer-Muller S, Shivakumar L, Fong K, Gao B, Randle D, Kondo M, Virmani A, Bader S, et al. (2001) Epigenetic inactivation of RASSF1A in lung and breast cancers and malignant phenotype suppression. *J. Natl. Cancer Inst*. **93**:691-699

Herman JG, Graff JR, Myohanen S, Nelkin BD, Baylin SB (1996) Methylation-specific PCR: a novel PCR assay for methylation status of CpG islands. *Proc. Natl. Acad. Sci. USA* **93**:9821-9826

Tsuchiya T, Tamura G, Sato K, Endoh Y, Sakata K, Jin Z, Motoyama T, Usuba O, Kimura W, Nishizuka S, et al. (2000) Distinct methylation patterns of two APC gene promoters in normal and cancerous gastric epithelia. *Oncogene* **19**:3642-3646

Virmani AK, Rathi A, Zochbauer-Muller S, Sacchi N, Fukuyama Y, Bryant D, Maitra A, Heda S, Fong KM, Thunnissen F, Minna JD, Gazdar AF (2000) Promoter methylation and silencing of the retinoic acid receptor-beta gene in lung carcinomas. *J. Natl. Cancer Inst*. **92**:1303-1307
